# Unusual complication following intramedullary nailing of tibia: a case report

**DOI:** 10.4076/1757-1626-2-7343

**Published:** 2009-09-14

**Authors:** Srinivasan Rajappa, N Sunil Kumar

**Affiliations:** Department of Orthopedics, Kumaran Hospital Pvt LtdPoonamalle High Road, Chennai - 600 006, Tamil NaduIndia

## Abstract

Intra-medullary nailing is a standard form of treatment for diaphyseal tibial fractures. Insertion of the nail requires threading of the nail over a smooth guide-wire. Inadvertent complications during this stage have been reported. We report a case of inadvertent penetration of the tibio-talar joint by the smooth guide wire caused by incarceration of a fracture fragment between the nail and the guide wire at the time of insertion. This was noted intra-operatively. The guide-wire and the nail were removed followed by insertion of a new nail and completion of the procedure. The patient did not have any symptoms attributable to this at the time of healing of her fracture.

## Case presentation

**Figure 1.**
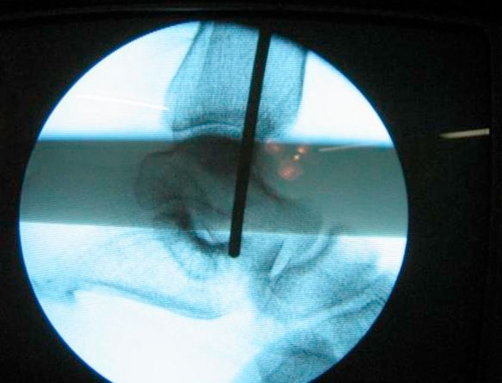
Guide-wire penetrating ankle and sub-talar joints. Intraoperative picture of the image intensifier screen showing penetration of the ankle and subtalar joint by the guidewire which was inserted.

**Figure 2.**
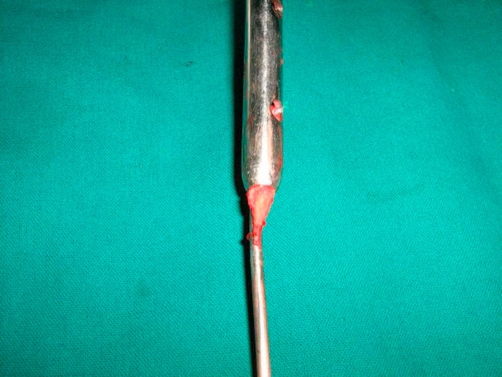
Incarcerated bone fragment between guidewire and intramedullary nail. Intraoperative picture of the incarceration of bone fragment between the guidewire and the intramedullary nail.

**Figure 3.**
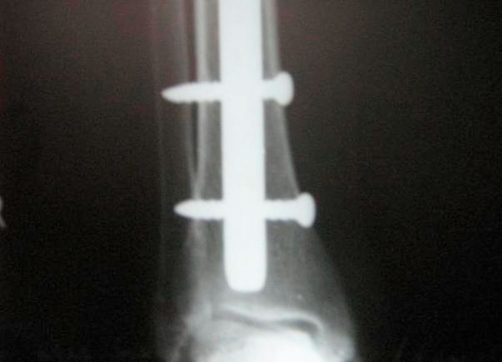
Completed nailing with interlocking bolts in position. Picture showing the distal locking bolts of the nail in place. This radiograph was taken at one and half years after initial procedure.

A 54 year old Sri Lankan woman was admitted for management of a grade 1 open fracture of both bones of her right leg. She also had a proximal humerus fracture on the left shoulder. After preliminary care in the emergency room, she was taken up for definitive surgical treatment of her fractures. The wound in her right leg was excised and primarily closed. This was followed by intra-medullary nailing of the tibia. A knobbed guide-wire was inserted through a patellar tendon-splitting approach through the tibial tubercle. This was followed by reaming of the medullary canal. The knobbed guide-wire was exchanged for a smooth guide wire followed by insertion of the nail. As the nail was hammered inside, a prominence was felt on the skin of the sole. Checking under image intensifier revealed that the guide-wire had penetrated the ankle and the sub-talar joint (Figure 1). The nail along with the guide-wire was removed and examined. This revealed incarceration of a fragment of bone between the guide-wire and nail preventing smooth sliding of the nail on the guide-wire (Figure 2). This had caused the nail and the guide-wire to move as a single piece resulting in penetration of the ankle and sub-talar joint by the guide-wire. A new guide-wire was inserted followed by completion of the nailing (Figure 3).

At the time of the last follow up, the patient did not have any residual ankle problems attributable to the complication.

## Discussion

Complications related to guide-wires have been reported during intra-medullary nailing [1]. Guide-wire penetration into the ankle and subtalar joint during reamed intramedullary nailing of tibia has been only once reported previously [2]. The authors reported a patient who presented with residual ankle pain following intramedullary nailing of the tibia. CT scans had revealed evidence of penetration of the talar dome by the guide-wire and also associated fractures of the talar body and calcaneus. The authors speculate that this could have happened at the time of reaming over a knobbed guide-wire without a bend proximal to the knob.

We have had a similar complication in our patient who underwent closed reamed intramedullary nailing of fracture of her tibia. However, our case was considerably different from the previous report. The penetration of the wire was detected intra-operatively when the assistant felt the guide wire in the sole of the patient’s foot. This had happened at the time of insertion of the nail over a straight guide-wire without any ball-tip (Figure 1). The nail and the wire were immediately removed and examination revealed incarceration of a piece of bone between the wire and the nail (Figure 2). This had prevented the smooth gliding of the nail over the guide-wire. Because of this the guide-wire-nail unit moved as single piece as the nail was hammered which resulted in the guide-wire penetrating the ankle and sub-talar joint. Our patient did not have any residual ankle problems at the time of fracture healing. This was probably related to the fact that the guide-wire was un-knobbed and hence the diameter of the hole in the tibial plafond, talus and calcaneus was small. In our patient there were not associated fractures of the Talus or Calcaenus as reported by Faraj *et al*.
